# Maternal central obesity and birth size: a Mendelian randomization analysis

**DOI:** 10.1186/s12944-018-0831-4

**Published:** 2018-07-31

**Authors:** Ting-Ting Geng, Tao Huang

**Affiliations:** 1 Saw Swee Hock School of Public Health, National University of Singapore, Singapore, 117549 Singapore; 2 Department of Epidemiology and Biostatistics, School of Public Health, Peking University Health Science Center, 38 Xueyuan Road, Haidian District, Beijing, 100191 China

**Keywords:** Maternal central obesity, Birth size, Birth weight, Birth length, Puberty height, Mendelian randomization

## Abstract

**Background:**

Observational studies have illustrated that maternal central obesity is associated with birth size, including of birth weight, birth length and head circumference, but the causal nature of these associations remains unclear. Our study aimed to test the causal effect of maternal central obesity on birth size and puberty height growth using a Mendelian randomization (MR) analysis.

**Methods:**

We performed two-sample MR using summary-level genome-wide public data. Thirty-five single nucleotide polymorphisms (SNPs), 25 SNPs and 41 SNPs were selected as instrumental variables for waist-to-hip ratio adjusted for BMI, waist circumference adjusted for BMI and hip circumference adjusted for BMI, respectively to test the causal effects of maternal central obesity on birth size and puberty height using an inverse-variance-weighted approach.

**Results:**

In this MR analysis, we found no evidence of a causal association between waist circumference or waist-to-hip ratio and the outcomes. However, we observed that one standard deviation (SD) increase in hip circumference (HIP) was associated with a 0.392 SD increase in birth length (*p* = 1.1 × 10^− 6^) and a 0.168 SD increase in birth weight (*p* = 7.1 × 10^− 5^), respectively at the Bonferroni-adjusted level of significance. In addition, higher genetically predicted maternal HIP was strongly associated with the puberty heights (0.835 SD, *p* = 8.4 × 10^− 10^). However, HIP was not associated with head circumference (*p* = 0.172).

**Conclusions:**

A genetic predisposition to higher maternal HIP was causally associated with larger offspring birth size independent of maternal BMI. However, we found no evidence of a causal association between maternal waist circumference, waist-to-hip ratio and birth size.

**Electronic supplementary material:**

The online version of this article (10.1186/s12944-018-0831-4) contains supplementary material, which is available to authorized users.

## Background

Overweight has become a world epidemic affecting women of childbearing age. Up to 50% of reproductive age women were overweight or obesity in Europe and the USA [1, 2]. Maternal overweight and obesity are associated with higher risks of many pregnancy complications and perinatal outcomes [3–6]. In addition, maternal overweight and obesity in pregnancy have been reported to be associated with early puberty development, such as earlier ages at menarche in daughters and earlier ages of voice break, acne and first nocturnal emission in sons [7, 8]. A previous Mendelian randomization study has illustrated that increased maternal BMI was causally associated with higher offspring birth weight [9].

However, waist circumference (WC), waist-to-hip ratio (WHR) and hip circumference (HIP), as indicators of central obesity have been suggested as being superior to BMI, given the relationship with visceral adiposity and the natural pregnant process [10–14]. Observational studies have documented that maternal central obesity is associated with an increased risk of adverse birth outcomes [15–18]. Nevertheless, maternal socioeconomic status and unmeasured lifestyle, such as smoking status, physical activity and diet might confound the observed associations. In addition, the inter-correlation of obesity traits also makes it difficult to examine the causal associations. Identifying a potential causal effect of maternal central obesity independent of BMI on birth size could clarify the causal association between maternal central obesity traits and birth size. Hence evidenced-based recommendations could be provided for pregnant women.

Mendelian Randomization (MR) analysis has become widely used to assess the potential causal relationship between environmental risk factors and diseases. MR studies have often been likened to natural randomized trials, in which genotype plays the role of random treatment assignment, avoiding the possibility of confounding and reverse causation [19–22]. It has been successfully explained the causal relationship between maternal BMI and birth weight [9]. Therefore, we conducted an MR analysis to investigate the relations of maternal central obesity with birth size and puberty heights using summary level data.

## Methods

### Study design

**Fig. 1:**
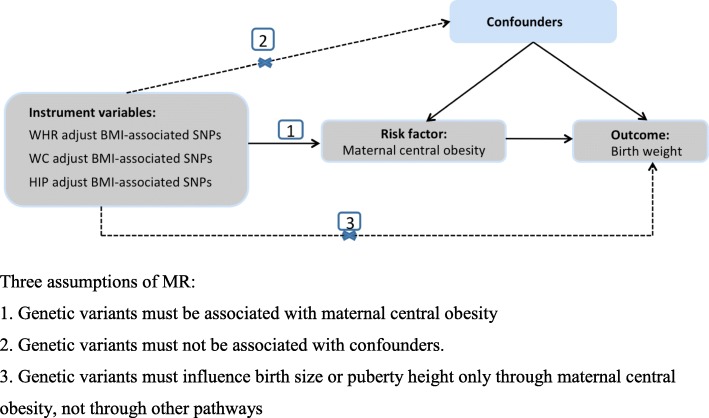
Schematic representation of a Mendelian randomization analysis

An MR analysis is free of confounding and reverse causation compared with observational studies. There are three assumptions of MR analysis (Fig. 1). First, the genetic variants used as instrumental variables (IVs) must be associated with maternal central obesity; second, the genetic variants must not be associated with any confounders; third, the genetic variants must be conditionally independent of the birth size and puberty height given the maternal central obesity and confounders of the risk factor-outcome relationship. The second and third assumptions are known as independence from pleiotropy [23, 24].

### Genetically predicted maternal central obesity

Genetically predicted maternal central obesity including of waist-to-hip ratio adjusted for BMI (WHRadjBMI), waist circumference adjusted for BMI (WCadjBMI) and hip circumference adjusted for BMI (HIPadjBMI) were based on single nucleotide polymorphisms (SNPs) of genome-wide significant (*P* < 5 × 10^− 8^) from the Genetic Investigation of Anthropometric Traits (GIANT) Consortium, which have been downloaded from http://portals.broadinstitute.org/collaboration/giant/index.php/GIANT_consortium_data_files.

GIANT consortium is an international collaboration that seeks to identify genetic loci that modulate human body size and shape, including height and measure of obesity. This genome-wide association meta-analysis (GWAS) included 224,459 individuals of European ancestry [25]. We assessed correlation (linkage disequilibrium) between SNPs using SNP Annotation and Proxy (SNAP) Search system (https://www.broadinstitute.org/snap/snap) for the same reference catalogue and population [26]. Highly correlated SNPs (*r*^2^ > 0.05) were discarded based on larger *P* value. We used 35 SNPs from GIANT consortium as an IV for WHRadjBMI, 25 SNPs for WCadjBMI and 41 SNPs for HIPadjBMI. Full details of the selected SNPs are provided in Additional file 1: Tables S1a, S1b and S1c. Any SNP for exposures not available for an outcome was replaced with a highly correlated proxy SNP (*r*^2^ > 0.8).

**Table 1:** Mendelian randomization analyses of maternal waist-to-hip ratio and birth size and puberty height

Outcomes	Consortium	Maternal WHRajdBMI (value of 1 SD with units, GIANT)							
		Main analysis		Sensitivity analysis					
		IVW		Weighted-median		MR-Egger			
		β (95% CI)	*P*-value	β (95% CI)	*P*-value	β (95% CI)	*P*-value	Intercept (95%CI)	*P*-value
Birth length(SD), cm									
Birth length	EGG	−0.01 (− 0.154 to 0.135)	0.896	− 0.03 (− 0.169 to 0.109)	0.675	−0.029 (− 0.583 to 0.524)	0.918	0.001 (− 0.02 to 0.021)	0.943
Birth weight(SD), kg									
Birth weight	EGG	−0.021 (− 0.095 to 0.053)	0.579	−0.015 (− 0.073 to 0.043)	0.622	0.142 (−0.136 to 0.419)	0.317	−0.006 (− 0.017 to 0.004)	0.232
Head circumference (HC)(SD), cm									
HC	EGG	−0.01 (− 0.154 to 0.135)	0.896	−0.03 (− 0.169 to 0.109)	0.675	−0.029 (− 0.583 to 0.524)	0.918	0.001 (− 0.02 to 0.021)	0.943
Puberty height single height measurement(SD), cm									
10F & 12 M	EGG	−0.10 (− 0.26 to 0.05)	0.207	−0.05 (− 0.24 to 0.14)	0.604	−0.23 (− 0.86 to 0.39)	0.466	0.005 (− 0.018 to 0.028)	0.678
10F	EGG	−0.064 (−0.267 to 0.137)	0.537	−0.09 (− 0.352 to 0.172)	0.499	−0.078 (− 0.843 to 0.687)	0.841	0.001 (−0.028 to 0.029)	0.97
12 M	EGG	−0.062 (−0.252 to 0.128)	0.529	−0.058 (− 0.317 to 0.201)	0.662	0.128 (−0.62 to 0.876)	0.738	−0.007 (− 0.035 to 0.02)	0.607

The threshold of significance was at the Bonferroni-adjusted level *P* < 0.0026 (0.05/35 = 0.00142)

*10F* Height SDS for females at age 10, *12 M* Height SDS for males at age 12, *10F&12 M* Height SDS for females at age 10 and males at age 12 combined

**Table 2:** Mendelian randomization analyses of maternal waist circumference and birth size and puberty height

Outcomes	Consortium	Maternal WCajdBMI (value of 1 SD with units, GIANT)							
		Main analysis		Sensitivity analysis					
		IVW		Weighted-median		MR-Egger			
		β (95% CI)	*P*-value	β (95% CI)	*P*-value	β (95% CI)	*P*-value	Intercept (95% CI)	*P*-value
Birth length(SD), cm									
Birth length	EGG	0.279 (0.095 to 0.464)	0.007	0.251 (0.057 to 0.446)	0.011	0.484 (−0.361 to 1.328)	0.262	−0.007 (− 0.036 to 0.022)	0.627
Birth weight(SD), kg									
Birth weight	EGG	0.114 (−0.028 to 0.255)	0.129	0.001 (−0.074 to 0.077)	0.977	0.1 (−0.524 to 0.723)	0.753	0.001 (−0.022 to 0.023)	0.964
Head circumference (HC)(SD),cm									
HC	EGG	−0.02 (− 0.199 to 0.159)	0.821	0.039 (−0.21 to 0.288)	0.76	−0.304 (−1.092 to 0.484)	0.45	0.01 (−0.016 to − 0.036)	0.452
Puberty height single height measurement(SD), cm									
10F & 12 M	EGG	0.345 (0.059 to 0.632)	0.027	0.358 (0.088 to 0.627)	0.009	0.359 (− 1.138 to 1.856)	0.638	0 (−0.051 to 0.05)	0.985
10F	EGG	0.379 (0.103 to 0.655)	0.013	0.305 (−0.018 to 0.628)	0.064	−0.132 (−1.371 to 1.108)	0.835	0.018(−0.025 to 0.061)	0.407
12 M	EGG	0.303 (−0.022 to 0.627)	0.081	0.194(− 0.171 to 0.558)	0.297	0.948 (−0.727 to 2.623)	0.267	−0.022 (− 0.078 to 0.034)	0.441

The threshold of significance was at the Bonferroni-adjusted level *P* < 0.0026 (0.05/25 = 0.002)

*10F* Height SDS for females at age 10, *12 M* Height SDS for males at age 12, *10F&12 M* Height SDS for females at age 10 and males at age 12 combined

**Table 3:** Mendelian randomization analyses of maternal hip circumference and birth size and puberty height

Outcomes	Consortium	Maternal HIPadjBMI (value of 1 SD with units, GIANT)							
		Main analysis		Sensitivity analysis					
		IVW		Weighted-median		MR-Egger			
		β (95% CI)	*P*-value	β (95% CI)	*P*-value	β (95% CI)	*P*-value	Intercept (95% CI)	*P*-value
Birth length(SD), cm									
Birth length	EGG	0.392(0.258 to 0.526)	1.12 × 10^−06^	0.433 (0.291 to 0.574)	2.2 × 10^−09^	0.798 (0.247 to 1.349)	0.005	−0.015 (− 0.036 to 0.005)	0.136
Birth weight(SD), kg									
Birth weight	EGG	0.168 (0.093 to 0.242)	7.1 × 10^−05^	0.175 (0.108 to 0.241)	2.5 × 10^−07^	0.153 (− 0.157 to 0.464)	0.332	0.001 (−0.011 to 0.012)	0.972
Head circumference (HC)(SD), cm									
HC	EGG	0.141 (−0.057 to 0.339)	0.172	0.111 (−0.106 to 0.328)	0.315	−0.149 (− 0.989 to 0.691)	0.728	0.011 (−0.02 to 0.042)	0.487
Puberty height single height measurement(SD), cm									
10F & 12 M	EGG	0.835 (0.631 to 1.038)	8.36 × 10^−10^	0.77 (0.556 to 0.985)	2.2 × 10^−12^	0.963 (0.086 to 1.841)	0.031	−0.005 (−0.037to 0.027)	0.768
10F	EGG	0.747 (0.452 to 1.041)	1.37 × 10^−05^	0.8 (0.506 to 1.094)	7.7 × 10^−08^	0.509 (− 0.758 to 1.775)	0.431	0.009 (−0.038 to 0.056)	0.705
12 M	EGG	0.828 (0.557 to 1.1)	5.63 × 10^−07^	0.834 (0.549 to 1.12)	1.0 × 10^− 08^	0.953 (− 0.218 to 2.125)	0.111	−0.005 (− 0.048 to 0.038)	0.83

The threshold of significance was at the Bonferroni-adjusted level *P* < 0.0026 (0.05/40 = 0.00125)

*10F* Height SDS for females at age 10, *12 M* Height SDS for males at age 12, *10F&12 M* Height SDS for females at age 10 and males at age 12 combined

### Genetically predicated birth size and puberty height

Genetic associations with birth weight [27], birth length [28], head circumference [29] and puberty growth [30] have been contributed by the Early Growth Genetics (EGG) Consortium from http://egg-consortium.org. The EGG Consortium represents a collaborative effort to combine data from multiple GWAS in order to identify additional human genome loci that have an impact on a variety of traits related to early growth. EGG birth weight data were imputed up to the reference panels from the 1000 Genomes Project (Phase 1 v 3) or combined 1000G and UK10K Project. Birth weight was z-score transformed in males and females separately. The birth weight dataset was generated by a European-ancestry GWAS meta-analysis (*n* = 153,781 individuals); the birth length, head circumference and puberty height growth datasets were generated by performing a meta-analysis of 22 European population-based studies (*n* = 28,459 individuals), a meta-analysis of 7 population-based European studies (*n* = 10,678 individuals) and a meta-analysis of 9 European cohort studies (*n* = 18,737), respectively.

### Statistical analysis

**Fig. 2:**
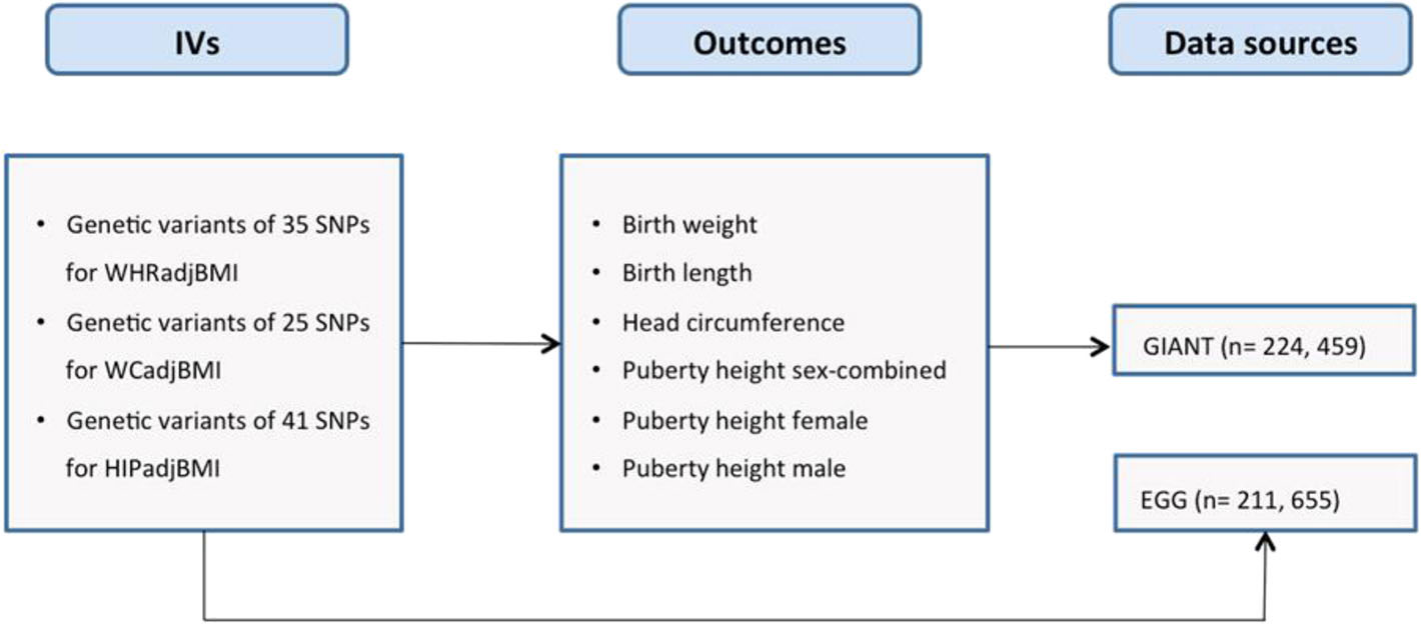
Data source of the Mendelian randomization analysis

The study design of the present MR was to explore the causal effect of maternal central obesity uponoffspring birth size (Fig. 2). SNPs were matched by assigning to the same effect allele firstly. Theestimates of the causal effect of maternal central obesity on birth size were analysed using the inversevarianceweighted (IVW). Provided that the genetic variants are uncorrelated, the IVW estimate is equivalentto a two-stage least squares analysis used with individual-level data. In IVW, the ratio estimates from eachIV are combined in an inverse-variance weighted estimator [21, 24]. From the analyses we reported themean difference for birth weight, birth length, head circumference and puberty height with 95% confidenceinterval (CI).

### Sensitivity analysis

Mendelian randomization analyses are becoming more powerful and efficient to perform based on summary public data. However, when using multiple genetic variants from different gene regions as IVs in an MR study, it is almost impossible that all the SNPs could meet the MR assumptions as mentioned previously [31]. Even if only one of the multiple genetic variants is an invalid instrumental variable, the casual effect will be overestimated [20]. In our study, we performed a series of sensitivity analyses to ensure the robust casual inferences.

If the causal association depends only on a single genetic variant and the estimate is heterogeneous with other variants’ estimates, then the results may be driven by a pleiotropic effect rather than the biology causal effect. We repeated the analyses excluding SNPs (26 SNPs left) that could influence WHRadjBMI and high-density lipoprotein cholesterol (HDL-C), triglycerides (TG), low-density lipoprotein cholesterol (LDL-C), adiponectin adjusted for BMI, fasting insulin adjusted for BMI, T2D, and height manually [25].

We also conducted sensitivity analyses to assess whether the estimates were robust to methodological choices. Weighted median method and MR-Egger regression were performed as complementary methods to ensure the robustness of the results. Weighted median estimate defines that each instrumental variable estimate appears with probability proportional to the inverse of its variance [32], which is consistent under the assumption that genetic variants, more than 50% of the weight in the analysis are valid instruments [21, 33].

MR-Egger regression based on Egger regression to examine publication bias in the meta-analysis was also used to test directional pleiotropy effects given no assumptions about the genetic variants but under an assumption that pleiotropic effects of genetic variants are independent of instrument strength. Using the MR-Egger regression method, the effect of IV on the exposure is plotted against its effect on the outcome, and an intercept distinct from the origin provides evidence for pleiotropic effects. The slope of the MR-Egger regression can provide pleiotropic-corrected causal estimates. If the intercept is zero, it suggests that there is no violation of the exclusion restriction criteria (i.e., no horizontal pleiotropy). It provides an estimate of the average pleiotropic effect across all of the genetic variants, because it reflects the effect of the joint instruments on outcome, when there is zero effect of the genetic variants of the risk factor [34–36].

Informed consent was obtained from all participants of contributing studies. Contributing studies received ethical approval from their respective institutional review boards. The statistical analyses were conducted using R version 3.2.4 (R Project for Statistical Computing). All statistical tests were 2-sided. The thresholds of statistical significance for WHRadjBMI, WCadjBMI and HIPadjBMI as exposures were *P* < 0.00142 (0.05/35 SNPs = 0.00142), *P* < 0.002 (0.05/25 SNPs = 0.002) and *P* < 0.00125 (0.05/41 SNPs = 0.00122), using Bonferroni test.

## Results

### Selected SNPs and instrumental variable validation

Based on the results of meta-analyses of GWAS, 35 SNPs, 25 SNPs and 41 SNPs reaching genome-wide significance for waist-to-hip ratio adjust for BMI, waist circumference adjust for BMI and hip circumference adjust for BMI were selected [25]. The characteristics of the selected SNPs for WHRadjBMI, WCadjBMI and HIPadjBMI selected are presented in Additional file 1: Tables S1a, S1b and S1c. None of the SNPs was found to be in LD with each other at an *r*^2^ > 0.05.

### Causal effects of WHRadjBMI, WCadjBMI on birth size and puberty height

We used the inverse-variance weighted method as the primary approach to examine the causal effect in the present MR study. Tables 1 and 2 show that genetically predicted WHRadjBMI and WCadjBMI were not associated with birth weight (beta,− 0.021,95% CI: − 0.095 to 0.053; beta, 0.114,95% CI: − 0.028 to 0.255), birth length (beta,− 0.01,95% CI: − 0.154 to 0.135; beta, 0.279,95% CI: 0.095 to 0.464), head circumference (beta,− 0.01,95% CI: − 0.154 to 0.135; beta,− 0.02,95% CI: − 0.199 to 0.159) or puberty height (beta,− 0.01,95% CI: − 0.26 to 0.05; beta, 0.354,95% CI: − 0.022 to 0.627). The results of the weighted median based method were consistent, illustrating non-causal effects of maternal WC on birth size or puberty height. The intercept term estimated from MR-Egger regression was centred at the origin with a confidence interval including the null showed that no evidence of directional horizontal pleiotropy effects.

### Causal effects of HIPadjBMI on birth size and puberty height

Table 3 shows that estimates for the causal effect of one SD increase in HIPadjBMI were consistently in the direction of 0.168 SD increase in birth weight (beta, 0.168, 95%CI: 0.093 to 0.242, *p* = 1.1 × 10^− 6^) and 0.392 SD increase in birth length (beta, 0.392, 95%CI: 0.258 to 0.526, *p* = 7.1 × 10^− 5^), respectively. We also found that one SD increase in HIPadjBMI was causally associated with a 0.835 SD increase in sex-combined puberty height (beta, 0.835, 95%CI: 0.631 to 1.038, *p* = 8.4 × 10^− 10^), a 0.747 SD increase in puberty height among females at 10 years old (beta, 0.747, 95%CI: 0.452 to 1.041, *p* = 1.4 × 10^− 5^) and a 0.828 SD increase in puberty height among males at 14 years old (beta, 0.828, 95%CI: 0.557 to 1.1, *p* = 5.6 × 10^− 7^). However, HIPadjBMI was not associated with head circumference (beta, 0.141, 95%CI: − 0.057 to 0.339). The results of the weighted median based method were consistent and even more significant than IVW method. The intercept term estimated from MR-Egger regression was centred at the origin with a confidence interval including the null showed that no evidence of pleiotropy effects.

### Sensitivity analyses of MR

We used the weighted median based method and MR-Egger method to estimate the causal effects of WHRadjBMI, WCadjBMI and HIPadjBMI on birth weight, birth length, head circumference and puberty height. The results consistently supported the robustness of our findings. The results of the causal effects of WHRadjBMI and outcomes were consistent with or without the 9 SNPs which had potentially pleiotropic effects.

## Discussion

In the present study, we used MR analyses to test the causal effect of maternal central obesity on offspring birth size and puberty height growth. We found that genetic predisposition to higher hip circumference independent of maternal BMI was associated with increased level of birth weight, birth length and puberty height. We observed one SD increase in HIPadjBMI (cm) was associated with 0.168 SD increase in birth weight, 0.392 SD increase in birth length and 0.835 SD increase in sex-combined puberty height. However, HIPadjBMI was not causally associated with head circumference. There was no evidence of causal effects of maternal WHRadjBMI and WCadjBMI on the birth size.

Observational studies have shown that maternal overweight and obesity were associated with many maternal and neonatal complications [37]. However, unmeasured or unknown confounding variables in the observational studies might have affected these associations. Socioeconomic factors and related behaviors such as smoking are critical confounders of observational associations between maternal central obesity and offspring birth size, since they are associated with both variables. In addition, the causal effects of maternal central obesity on birth size could also be confounded by maternal BMI. Therefore, the causality of these observations remains unclear. The IVs used in this MR were genetic variants associated with maternal central obesity adjusted for BMI, which could avoid the socioeconomic, behavior factors and BMI confounding, since genotypes are determined at conception.

The findings from this study were inconsistent with the results from the observational studies that showed women with increased WHR or WC were more likely to give birth to macrosomia [16, 38]. Li et al. reported that WC was positively associated with risk of macrosomia (OR, 1.58; 95%CI, 1.07–2.32) [16]. Salem et al. showed that the risk of macrosomia was 1.7 times higher in fourth quartiles WHR [38]. Piperata et al. illustrated women who had normal birth weight infants showed substantial positive association with HIP [39], which corroborates with our findings.

The pathophysiological mechanisms underlying the causation relationship between HIP and birth size is not fully understood. The potential mechanism could be explained below. Larger HIP potentially reflects increased gluteofemoral muscle mass. As muscle mass increases with weight even in the overweight and obese women, HIP increases with WC as well. The side effects of larger WC might be counteracted by the increased muscle mass to some extent [40]. In addition, maternal lean body mass has been illustrated to be one of the major determinants of birth weight [41, 42]. Whereas WC and WHR are predictors of abdominal adiposity, which were reported to be substantially associated with macrosomia [38]. Maternal visceral obesity was found to impair the anabolic response and the activity of SNAT, which is associated with fetal growth, such as skeletal growth or lean mass growth and final birth size [43, 44]. In additional maternal larger WC or WHR is associated with a higher risk of gestational weight gain, which has been reported to increase the risk of high birth weight, macrosomia and large-for-gestational-age infants [45]. WC and WHR seem to be more sensitive to the low birth weight (< 2.5 kg) or macrosomia (> 4 kg). The mean birth weight included in the cohort studies in EGG consortia that used in our MR were ranged from 3.0 kg to 3.7 kg, which was the normal birth weight according to the birth weight cut off (2.5 kg–4 kg) [27]. Therefore, in our MR study, HIP, rather than WC or WHR was causally associated with birth weight.

### Strength and limitations

Our large two sample MR study using public data provided more precise estimates with greater statistical power because of the large sample size. The consistency significant causal effects estimates from different approaches showed no evidence of a violation of the MR assumption and suggested robustness of our findings. Additionally, we used three genetic IVs as indicators of central obesity (WHRadjBMI, WCadjBMI and HIPadjBMI) to test the associations of maternal central obesity independent of maternal BMI and offspring birth size and puberty height. However, there are several limitations of our study. Firstly, we used two-sample MR to explore the causal effect of maternal exposure on later offspring outcomes. Though in theory two-sample MR could be used to explain this issue; in general it is impossible to assess many questions related to intrauterine effects on offspring outcomes. Therefore, individual participant data of maternal genetic variants on offspring outcomes are warranted replicating the results. Second, the causal relationship between maternal hip circumference and offspring birth size could be violated via the offspring’s genetic variants [46]. This issue would be more serious in the situation when the maternal exposure and offspring outcomes are the same characteristic or very similar. Our study aimed to investigate the causal effect of maternal central obesity and offspring birth weight; it is plausible that there would be some overlap between the maternal genetic IVs and offspring genetic IVs. Due to our summary data MR study design, it is impossible to adjust offspring IVs in our study. Further MR is warranted in the use of individual data. Third, we used genetic variants that have been shown to be robustly related to the exposure in GWAS conducted in non-pregnant women. The critical question here is whether genetic variants identified in non-pregnant women are valid IVs for pregnancy exposures. However, it has been illustrated that for some genetic variants, associations with exposures measured in pregnancy are similar to those in GWAS of non-pregnant women [9, 47]. Fourth, MR studies are often investigating the causal effect of the life cumulative exposure on outcomes. In this study, we addressed the specific question in the specific time period, intrauterine period. The causal effects on birth size could be biased by the pre or post pregnancy maternal central obesity [48]. In addition, we assumed that the association between maternal central obesity and birth size was linear. If the relationship was non-linear, it is necessary for the association of the IV with the exposure in the population to remain constant at different levels of the exposure [31]. However, deviations from the assumption would result in reduced statistical power in risk analyses, rather than generating spurious associations. Finally, our study was restricted to individuals of European ancestry; the association of genetic HIP adjusted for BMI with birth weight may differ by ethnicity or genetic ancestry. Our results may not be generalized to non-European populations.

## Conclusions

In this MR study, a genetic predisposition to higher maternal hip circumference was potentially causally associated with higher offspring birth weight, birth height and puberty height.

## Additional file

Additional file 1: **Table S1a.** WHRadjBMI loci achieving genome-wide significance (*P* < 5 × 10^–8^) in European women-specific meta-analyses. **Table S1b.** WCadjBMI loci achieving genome-wide significance (*P* < 5 × 10^–8^) in European women-specific meta-analyses. **Table S1c.** HIPadjBMI loci achieving genome-wide significance (*P* < 5 × 10^–8^) in European women-specific meta-analyses. (XLSX 22 kb)

## Abbreviations

EGG: Early Growth Genetics
GIANT: Genetic Investigation of Anthropometric Traits
GWAS: Genome-wide association meta-analysis
HDL-C: High-density lipoprotein cholesterol
HIP: Hip circumference
HIPadjBMI: Hip circumference adjusted for BMI
IV: Instrumental variable
IVW: Inverse-variance weighted
LDL-C: Low-density lipoprotein cholesterol
MR: Mendelian randomization
SD: Standard deviation
SNAP: SNP Annotation and Proxy
SNPs: Single nucleotide polymorphisms
T2DM: Type 2 diabetes mellitus
TG: Triglycerides
WC: Waist circumference
WCadjBMI: Waist circumference adjusted for BMI
WHR: Waist-to-hip ratio
WHRadjBMI: Waist-to-hip ratio adjusted for BMI

## References

[1] Li C, Ford ES, McGuire LC, Mokdad AH, Little RR, Reaven GM (2006). Trends in hyperinsulinemia among nondiabetic adults in the U.S. Diabetes Care.

[2] Heslehurst N, Ells LJ, Simpson H, Batterham A, Wilkinson J, Summerbell CD (2007). Trends in maternal obesity incidence rates, demographic predictors, and health inequalities in 36,821 women over a 15-year period. BJOG.

[3] Miao M, Dai M, Zhang Y, Sun F, Guo X, Sun G (2017). Influence of maternal overweight, obesity and gestational weight gain on the perinatal outcomes in women with gestational diabetes mellitus. Sci Rep.

[4] Lawlor DA, Fraser A, Lindsay RS, Ness A, Dabelea D, Catalano P, Davey Smith G, Sattar N, Nelson SM (2010). Association of existing diabetes, gestational diabetes and glycosuria in pregnancy with macrosomia and offspring body mass index, waist and fat mass in later childhood: findings from a prospective pregnancy cohort. Diabetologia.

[5] Schmatz M, Madan J, Marino T, Davis J (2010). Maternal obesity: the interplay between inflammation, mother and fetus. J Perinatol.

[6] Harmon KA, Gerard L, Jensen DR, Kealey EH, Hernandez TL, Reece MS, Barbour LA, Bessesen DH (2011). Continuous glucose profiles in obese and normal-weight pregnant women on a controlled diet: metabolic determinants of fetal growth. Diabetes Care.

[7] Hounsgaard ML, Hakonsen LB, Vested A, Thulstrup AM, Olsen J, Bonde JP, Nohr EA, Ramlau-Hansen CH (2014). Maternal pre-pregnancy body mass index and pubertal development among sons. Andrology.

[8] Deardorff J, Berry-Millett R, Rehkopf D, Luecke E, Lahiff M, Abrams B (2013). Maternal pre-pregnancy BMI, gestational weight gain, and age at menarche in daughters. Matern Child Health J.

[9] Tyrrell J, Richmond RC, Palmer TM, Feenstra B, Rangarajan J, Metrustry S, Cavadino A, Paternoster L, Armstrong LL, De Silva NM (2016). Genetic evidence for causal relationships between maternal obesity-related traits and birth weight. JAMA.

[10] Povel CM, Beulens JW, van der Schouw YT, Dolle ME, Spijkerman AM, Verschuren WM, Feskens EJ, Boer JM (2013). Metabolic syndrome model definitions predicting type 2 diabetes and cardiovascular disease. Diabetes Care.

[11] Liu PY, Hornbuckle LM, Panton LB, Kim JS, Ilich JZ (2012). Evidence for the association between abdominal fat and cardiovascular risk factors in overweight and obese African American women. J Am Coll Nutr.

[12] Hidayat K, Du X, Chen G, Shi M, Shi B (2016). Abdominal obesity and lung cancer risk: systematic review and meta-analysis of prospective studies. Nutrients..

[13] Aune D, Greenwood DC, Chan DS, Vieira R, Vieira AR, Navarro Rosenblatt DA, Cade JE, Burley VJ, Norat T (2012). Body mass index, abdominal fatness and pancreatic cancer risk: a systematic review and non-linear dose-response meta-analysis of prospective studies. Ann Oncol.

[14] Lee CM, Huxley RR, Wildman RP, Woodward M (2008). Indices of abdominal obesity are better discriminators of cardiovascular risk factors than BMI: a meta-analysis. J Clin Epidemiol.

[15] Cisneiros RM, Dutra LP, Silveira FJ, Souza AR, Marques M, Amorim MM, Urquia ML, Ray JG, Alves JG (2013). Visceral adiposity in the first half of pregnancy predicts newborn weight among adolescent mothers. J Obstet Gynaecol Can.

[16] Li S, Rosenberg L, Palmer JR, Phillips GS, Heffner LJ, Wise LA (2013). Central adiposity and other anthropometric factors in relation to risk of macrosomia in an African American population. Obesity (Silver Spring).

[17] Suresh A, Liu A, Poulton A, Quinton A, Amer Z, Mongelli M, Martin A, Benzie R, Peek M, Nanan R (2012). Comparison of maternal abdominal subcutaneous fat thickness and body mass index as markers for pregnancy outcomes: a stratified cohort study. Aust N Z J Obstet Gynaecol.

[18] Nehring I, Chmitorz A, Reulen H, von Kries R, Ensenauer R (2013). Gestational diabetes predicts the risk of childhood overweight and abdominal circumference independent of maternal obesity. Diabet Med.

[19] Neeland IJ, Kozlitina J (2017). Mendelian randomization: using natural genetic variation to assess the causal role of modifiable risk factors in observational studies. Circulation.

[20] Burgess S, Thompson SG (2013). Use of allele scores as instrumental variables for Mendelian randomization. Int J Epidemiol.

[21] Burgess S, Butterworth A, Thompson SG (2013). Mendelian randomization analysis with multiple genetic variants using summarized data. Genet Epidemiol.

[22] Lawlor DA, Harbord RM, Sterne JA, Timpson N, Davey Smith G (2008). Mendelian randomization: using genes as instruments for making causal inferences in epidemiology. Stat Med.

[23] Thompson JR, Minelli C, Del Greco MF (2016). Mendelian randomization using public data from genetic consortia. Int J Biostat.

[24] Burgess S, Scott RA, Timpson NJ, Davey Smith G, Thompson SG (2015). Using published data in Mendelian randomization: a blueprint for efficient identification of causal risk factors. Eur J Epidemiol.

[25] Shungin D, Winkler TW, Croteau-Chonka DC, Ferreira T, Locke AE, Magi R, Strawbridge RJ, Pers TH, Fischer K, Justice AE (2015). New genetic loci link adipose and insulin biology to body fat distribution. Nature.

[26] Johnson AD, Handsaker RE, Pulit SL, Nizzari MM, O’Donnell CJ, de Bakker PI (2008). SNAP: a web-based tool for identification and annotation of proxy SNPs using HapMap. Bioinformatics.

[27] Horikoshi M, Beaumont RN, Day FR, Warrington NM, Kooijman MN, Fernandez-Tajes J, Feenstra B, van Zuydam NR, Gaulton KJ, Grarup N (2016). Genome-wide associations for birth weight and correlations with adult disease. Nature.

[28] van der Valk RJ, Kreiner-Moller E, Kooijman MN, Guxens M, Stergiakouli E, Saaf A, Bradfield JP, Geller F, Hayes MG, Cousminer DL (2015). A novel common variant in DCST2 is associated with length in early life and height in adulthood. Hum Mol Genet.

[29] Taal HR, St Pourcain B, Thiering E, Das S, Mook-Kanamori DO, Warrington NM, Kaakinen M, Kreiner-Moller E, Bradfield JP, Freathy RM (2012). Common variants at 12q15 and 12q24 are associated with infant head circumference. Nat Genet.

[30] Cousminer DL, Berry DJ, Timpson NJ, Ang W, Thiering E, Byrne EM, Taal HR, Huikari V, Bradfield JP, Kerkhof M (2013). Genome-wide association and longitudinal analyses reveal genetic loci linking pubertal height growth, pubertal timing and childhood adiposity. Hum Mol Genet.

[31] Burgess S, Bowden J, Fall T, Ingelsson E, Thompson SG (2017). Sensitivity analyses for robust causal inference from Mendelian randomization analyses with multiple genetic variants. Epidemiology.

[32] Bowden J, Davey Smith G, Haycock PC, Burgess S (2016). Consistent estimation in Mendelian randomization with some invalid instruments using a weighted median estimator. Genet Epidemiol.

[33] Yavorska OO, Burgess S (2017). MendelianRandomization: an R package for performing Mendelian randomization analyses using summarized data. Int J Epidemiol..

[34] Bowden J, Davey Smith G, Burgess S (2015). Mendelian randomization with invalid instruments: effect estimation and bias detection through egger regression. Int J Epidemiol.

[35] Bowden J, Del Greco MF, Minelli C, Davey Smith G, Sheehan N, Thompson J (2017). A framework for the investigation of pleiotropy in two-sample summary data Mendelian randomization. Stat Med.

[36] Bowden J, Del Greco MF, Minelli C, Davey Smith G, Sheehan NA, Thompson JR (2016). Assessing the suitability of summary data for two-sample Mendelian randomization analyses using MR-egger regression: the role of the I2 statistic. Int J Epidemiol.

[37] Djelantik AA, Kunst AE, van der Wal MF, Smit HA, Vrijkotte TG (2012). Contribution of overweight and obesity to the occurrence of adverse pregnancy outcomes in a multi-ethnic cohort: population attributive fractions for Amsterdam. BJOG.

[38] Salem W, Adler AI, Lee C, Smith GC (2012). Maternal waist to hip ratio is a risk factor for macrosomia. BJOG.

[39] Piperata BA, Dufour DL, Reina JC, Spurr GB (2002). Anthropometric characteristics of pregnant women in Cali, Colombia and relationship to birth weight. Am J Hum Biol.

[40] Conway B, Xiang YB, Villegas R, Zhang X, Li H, Wu X, Yang G, Gao YT, Zhang W, Shu XO (2011). Hip circumference and the risk of type 2 diabetes in middle-aged and elderly men and women: the shanghai women and shanghai men’s health studies. Ann Epidemiol.

[41] Kulkarni B, Shatrugna V, Balakrishna N (2006). Maternal lean body mass may be the major determinant of birth weight: a study from India. Eur J Clin Nutr.

[42] Mongelli M (1996). Maternal lean body mass and birth-weight. Aust N Z J Obstet Gynaecol.

[43] Duggleby SL, Jackson AA (2001). Relationship of maternal protein turnover and lean body mass during pregnancy and birth length. Clin Sci (Lond).

[44] Farley DM, Choi J, Dudley DJ, Li C, Jenkins SL, Myatt L, Nathanielsz PW (2010). Placental amino acid transport and placental leptin resistance in pregnancies complicated by maternal obesity. Placenta.

[45] Lawlor DA, Relton C, Sattar N, Nelson SM (2012). Maternal adiposity--a determinant of perinatal and offspring outcomes?. Nat Rev Endocrinol.

[46] Lawlor D, Richmond R, Warrington N, McMahon G, Davey Smith G, Bowden J, Evans DM (2017). Using Mendelian randomization to determine causal effects of maternal pregnancy (intrauterine) exposures on offspring outcomes: sources of bias and methods for assessing them. Wellcome Open Res.

[47] Cho YM, Kim TH, Lim S, Choi SH, Shin HD, Lee HK, Park KS, Jang HC (2009). Type 2 diabetes-associated genetic variants discovered in the recent genome-wide association studies are related to gestational diabetes mellitus in the Korean population. Diabetologia.

[48] Lawlor DA, Tilling K, Davey Smith G (2016). Triangulation in aetiological epidemiology. Int J Epidemiol.

